# Genome-wide local ancestry and evidence for mitonuclear coadaptation in African hybrid cattle populations

**DOI:** 10.1016/j.isci.2022.104672

**Published:** 2022-06-26

**Authors:** James A. Ward, Gillian P. McHugo, Michael J. Dover, Thomas J. Hall, Said Ismael Ng'ang'a, Tad S. Sonstegard, Daniel G. Bradley, Laurent A.F. Frantz, Michael Salter-Townshend, David E. MacHugh

**Affiliations:** 1 Animal Genomics Laboratory, UCD School of Agriculture and Food Science, University College Dublin, Belfield, D04 V1W8 Dublin, Ireland; 2 Palaeogenomics Group, Department of Veterinary Sciences, Ludwig Maximilian University, 80539 Munich, Germany; 3 School of Biological and Chemical Sciences, Queen Mary University of London, London E1 4NS, UK; 4 Acceligen, 3388 Mike Collins Drive, Eagan, MN 55121, USA; 5 Smurfit Institute of Genetics, Trinity College Dublin, D04 V1W8 Dublin, Ireland; 6 UCD School of Mathematics and Statistics, University College Dublin, D04 V1W8 Dublin, Ireland; 7 UCD Conway Institute of Biomolecular and Biomedical Research, University College Dublin, D04 V1W8 Dublin, Ireland

**Keywords:** Ecology, Biological sciences, Genetics, Genomics, Evolutionary biology

## Abstract

The phenotypic diversity of African cattle reflects adaptation to a wide range of agroecological conditions, human-mediated selection preferences, and complex patterns of admixture between the humpless *Bos taurus* (taurine) and humped *Bos indicus* (zebu) subspecies, which diverged 150–500 thousand years ago. Despite extensive admixture, all African cattle possess taurine mitochondrial haplotypes, even populations with significant zebu biparental and male uniparental nuclear ancestry. This has been interpreted as the result of human-mediated dispersal ultimately stemming from zebu bulls imported from South Asia during the last three millennia. Here, we assess whether ancestry at mitochondrially targeted nuclear genes in African admixed cattle is impacted by mitonuclear functional interactions. Using high-density SNP data, we find evidence for mitonuclear coevolution across hybrid African cattle populations with a significant increase of taurine ancestry at mitochondrially targeted nuclear genes. Our results, therefore, support the hypothesis of incompatibility between the taurine mitochondrial genome and the zebu nuclear genome.

## Highlights

• Using SNP data, we find evidence for mitonuclear coevolution in hybrid African cattle
• Increased taurine ancestry at mitochondrially targeted nuclear genes in several breeds
• Observed for genes encoding proteins that interact with mtDNA-encoded proteins
• Our results support the hypothesis of mitonuclear incompatibility in African cattle

## Graphical abstract

**Figure:**
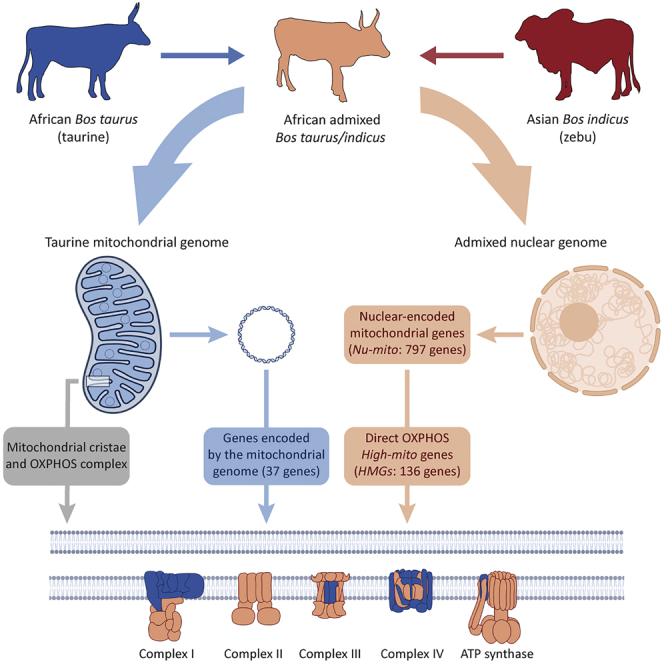

## Introduction

Hybridization between divergent lineages results in an influx of new genetic variants which can improve the adaptive potential of animal and plant populations (Hedrick, 2013; Moran et al., 2021). It has long been used by breeders to generate livestock populations with specific phenotypic characteristics (Wu and Zhao, 2021). For example, crossbreeding between Asian and European domestic pigs, which diverged ∼1 million year ago, was used by 19^th^-century European breeders as a strategy to improve the fertility of local landraces (Bosse et al., 2014; White, 2011).

Human-mediated crossbreeding between the humpless *Bos primigenius taurus* (*B. taurus* – taurine) and the humped *Bos primigenius indicus* (*B. indicus* – zebu), which diverged 150–500 kya (Chen et al., 2018; Wang et al., 2018; Wu et al., 2018), has also played a major role in shaping the genetic composition of many African cattle populations. In fact, recent nuclear genome studies have shown that cattle ancestry in Africa represents a mosaic shaped by admixture between the original substrate of locally adapted taurine cattle, which likely first came to Africa with people during the Neolithic period, and the more recently introduced South Asian zebu (Kim et al., 2017, 2020). This process of admixture, which lasted around 2,000 years (Kim et al., 2020), led to the establishment of indigenous African cattle populations that are deeply rooted in rural African communities, forming an integral part of food production and cultural and religious activities throughout the continent (Van Marle-Köster et al., 2021). The establishment of these African cattle populations with their unique phenotypic adaptations has been influenced by specific livestock breeding practices. In addition, their biology has been influenced by adaption to savanna biomes, cultural preferences, the logistics of long-distance terrestrial and maritime trade networks encompassing southern Asia, Arabia, and North and East Africa (Boivin et al., 2014; Boivin and Fuller, 2009; Gifford-Gonzalez and Hanotte, 2011; Marshall, 1989), and the massive cattle replacements following the rinderpest panzootics of the late 19th century (Spinage, 2003).

Despite extensive admixture, however, all mitochondrial genomes of native African cattle populations analyzed to date exclusively cluster within the taurine T1 haplogroup (Bradley et al., 1996; Kwon et al., 2022; Loftus et al., 1994a, 1994b; Troy et al., 2001). This observation, together with the widespread distribution of *B. indicus* Y chromosome haplotypes across Africa (Hanotte et al., 2000; Perez-Pardal et al., 2018), has been interpreted as the result of human-mediated dispersal and breeding of zebu bulls from South Asia during the last three millennia (Hanotte et al., 2000, 2002; MacHugh et al., 1997; Perez-Pardal et al., 2018).

Functional mismatches between the mitochondrial and nuclear genomes transmitted from two divergent parental lineages have been observed in many vertebrate populations (Hill, 2019; Hill et al., 2019). For example, recent studies on hybridization in cattle, hares, sparrows, and hominids have provided compelling evidence for mitonuclear incompatibilities (Kwon et al., 2022; Seixas et al., 2018; Sharbrough et al., 2017; Trier et al., 2014). These likely stem from the fact that the 37 genes located in vertebrate mitochondrial genomes (Boore, 1999) also rely on over one thousand coadapted nuclear genes that encode proteins and protein subunits essential to the efficient functioning of the mitochondrion (Blier et al., 2001; Rand et al., 2004; Sloan et al., 2018; Woodson and Chory, 2008). The most well-studied example of mitonuclear cooperation is the oxidative phosphorylation (OXPHOS) system, which consists of five protein complexes, four of which are chimeric—assembled using subunits encoded by both the nuclear and mitochondrial genomes (Allen, 2015; Isaac et al., 2018; Rand et al., 2004). Mitonuclear incompatibilities between distinct inter- and intraspecific evolutionary lineages can give rise to deleterious biochemical effects associated with reduced efficacy of OXPHOS protein complexes (Ballard and Melvin, 2010; Blier et al., 2001; Ellison and Burton, 2006; Ellison et al., 2008), which lead to lower ATP production (Ellison and Burton, 2006; Ellison et al., 2008; McKenzie et al., 2003, 2004) and increased levels of oxidative damage (Barreto and Burton, 2013; Du et al., 2017; Latorre-Pellicer et al., 2016; Pichaud et al., 2019).

**Figure 1:**
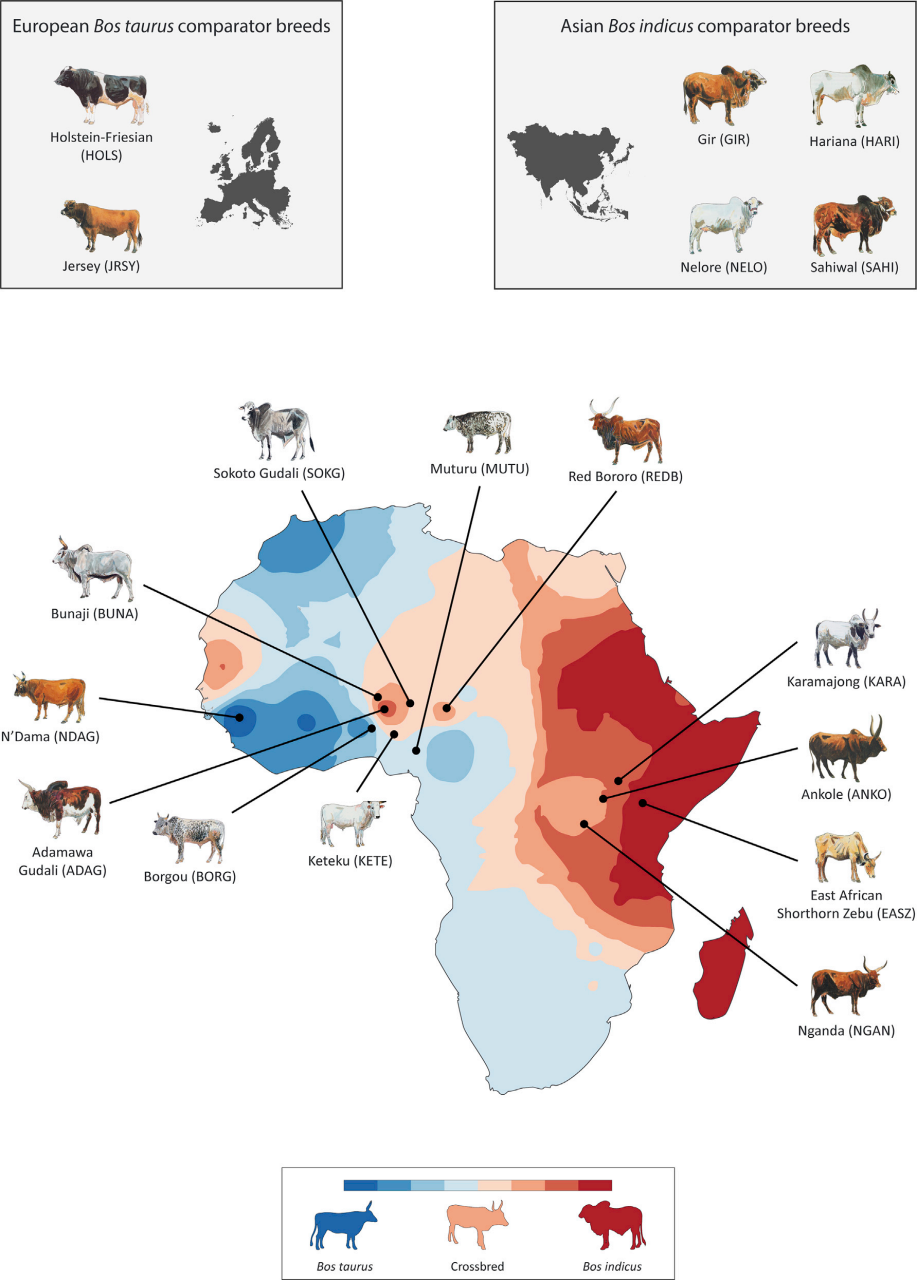
Geographical patterns of African *Bos taurus* and Asian *Bos indicus* admixture in hybrid African cattle populations Map of Africa showing sampled cattle populations and an interpolated synthetic map illustrating spatial distribution of African *Bos taurus* and Asian *Bos indicus* admixture. Also shown are two European *B. taurus* and four Asian *B. indicus* comparator breeds. Admixture data were generated from the first principal component (PC1) of a principal component analysis (PCA) of microsatellite genetic variation across African cattle populations (Hanotte et al., 2002). Modified from McHugo et al. (2019) under the terms of the Creative Commons Attribution 4.0 International License (http://creativecommons.org/licenses/by/4.0). Individual cattle art images modified from Felius (1995) with permission of the author.

Fixation of the T1 haplogroup in African cattle has been investigated recently. An approximate Bayesian computation approach using genome-wide nuclear SNP data from 162 East African cattle indicated that a model of male-mediated dispersal combined with mitonuclear interactions could explain the current patterns of bovine genomic diversity in this region (Kwon et al., 2022). Here, we examine discordance of uniparental and biparental genomic variation in African cattle and test the hypothesis that functional incompatibilities have arisen between the mitochondrial and nuclear genomes in hybrid cattle populations across the continent (Figure 1). To do this, we analyzed high-density SNP data encompassing the nuclear and mtDNA genomes (Illumina BovineHD 777K BeadChip) from 605 animals representing 18 African, Asian, and European breeds/populations and 174 complete bovine mitochondrial genomes. These data were used to characterize genome-wide local ancestry and systematically evaluate mitonuclear interactions, coadaptation, and functional mismatch in multiple genetically independent admixed African cattle populations.

## Results and discussion

### Complex mitonuclear genomic structure in African admixed cattle

**Figure 2:**
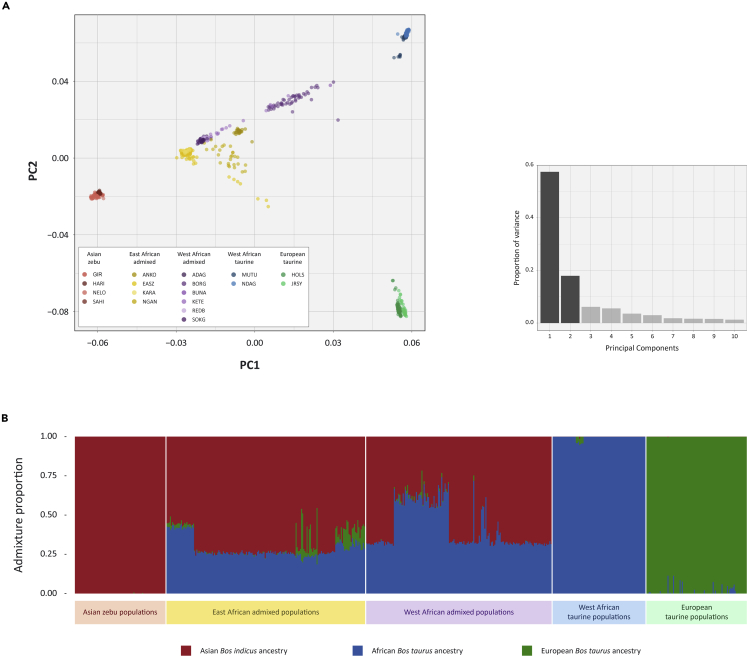
Autosomal genomic diversity and admixture in African, Asian, and European cattle (A) Results of the principal component analysis (PCA) for 605 animals from 18 different cattle breeds genotyped for 562,635 SNPs. The PCA plot shows the coordinates for each animal based on the first two principal components. Principal component 1 (PC1) differentiates the *Bos taurus* and *Bos indicus* evolutionary lineages, whereas PC2 separates the African and European taurine groups. A histogram plot of the relative variance contributions for the first 10 PCs is also shown with PC1 and PC2 accounting for 58.4 and 17.9% of the total variation for PC1–10, respectively. (B) Unsupervised genetic structure plot for Asian zebu, East and West African admixed cattle, and West African and European taurine breeds. Results for an inferred number of ancestry clusters of K = 3 is shown, which corresponds to Asian *Bos indicus* (red), European *Bos taurus* (green), and African *B. taurus* (blue) ancestral components, respectively.

We first established the ancestry of the animals in our sample set using the BovineHD 777K BeadChip data. Filtering and quality control of the BovineHD 777K BeadChip resulted in 562,635 SNPs and 605 individual animals, retained for subsequent analyses (Table 1). Figure 2A shows a principal-component analysis (PCA) plot generated using SNP genotype data for Asian *B. indicus*, East and West African admixed *B. indicus/taurus*, African *B. taurus*, and European *B. taurus* cattle. PC1 (58.4%) and PC2 (17.9%) account for the bulk of the variance and represent the splits between *B. indicus* and *B. taurus* and the African and European taurine lineages, respectively. The results of the genetic structure analysis using the fastSTRUCTURE program and an inferred number of clusters of *K* = 3 are shown in Figure 2B, which illustrates taurine and zebu autosomal genomic ancestry across individual East and West African admixed animals and breeds (Figure S1 and Table S1). These results recapitulate, at higher resolution, the continent-wide patterns of admixture that were previously observed using smaller panels of microsatellite and SNP markers (Decker et al., 2014; Hanotte et al., 2002).

**Table 1:** Cattle breeds/populations, geographical origins, and sources of BovineHD 777K SNP data

Cattle breed/population	Code	Type/morphology	Country of origin	Source 1^a^	Source 2^b^	Source 3^c^	Source 4^d^	Total (*n*)
Muturu	MUTU	West African taurine	Nigeria	8	–	20	–	28
N’Dama	NDAG	West African taurine	Guinea	24	9	–	23	56
Holstein-Friesian	HOLS	European taurine	Netherlands	59	–	–	–	59
Jersey	JRSY	European taurine	United Kingdom	32	–	–	–	32
Ankole	ANKO	East African admixed	Uganda	25	–	–	–	25
East African Shorthorn Zebu	EASZ	East African admixed	Kenya	92	–	19	–	111
Karamojong	KARA	East African admixed	Uganda	16	–	–	–	16
Nganda	NGAN	East African admixed	Uganda	23	–	4	–	27
Adamawa Gudali	ADAG	West African admixed	Nigeria	23	–	2	–	25
Borgou	BORG	West African admixed	Benin	–	–	–	50	50
Bunaji	BUNA	West African admixed	Nigeria	22	–	5	–	27
Keteku	KETE	West African admixed	Nigeria	–	–	22	–	22
Red Bororo	REDB	West African admixed	Nigeria	22	–	4	–	26
Sokoto Gudali	SOKG	West African admixed	Nigeria	19	–	–	–	19
Gir	GIR	Asian zebu	India	28	–	–	–	28
Hariana	HARI	Asian zebu	India	–	10	–	–	10
Nelore	NELO	Asian zebu	Brazil	34	–	–	–	34
Sahiwal	SAHI	Asian zebu	India	–	10	–	–	10
**Total**								**605**

a (Bahbahani et al., 2017).

b Verdugo et al. (2019).

c Samples genotyped by Acceligen.

d genotyped for the present study.

**Figure 3:**
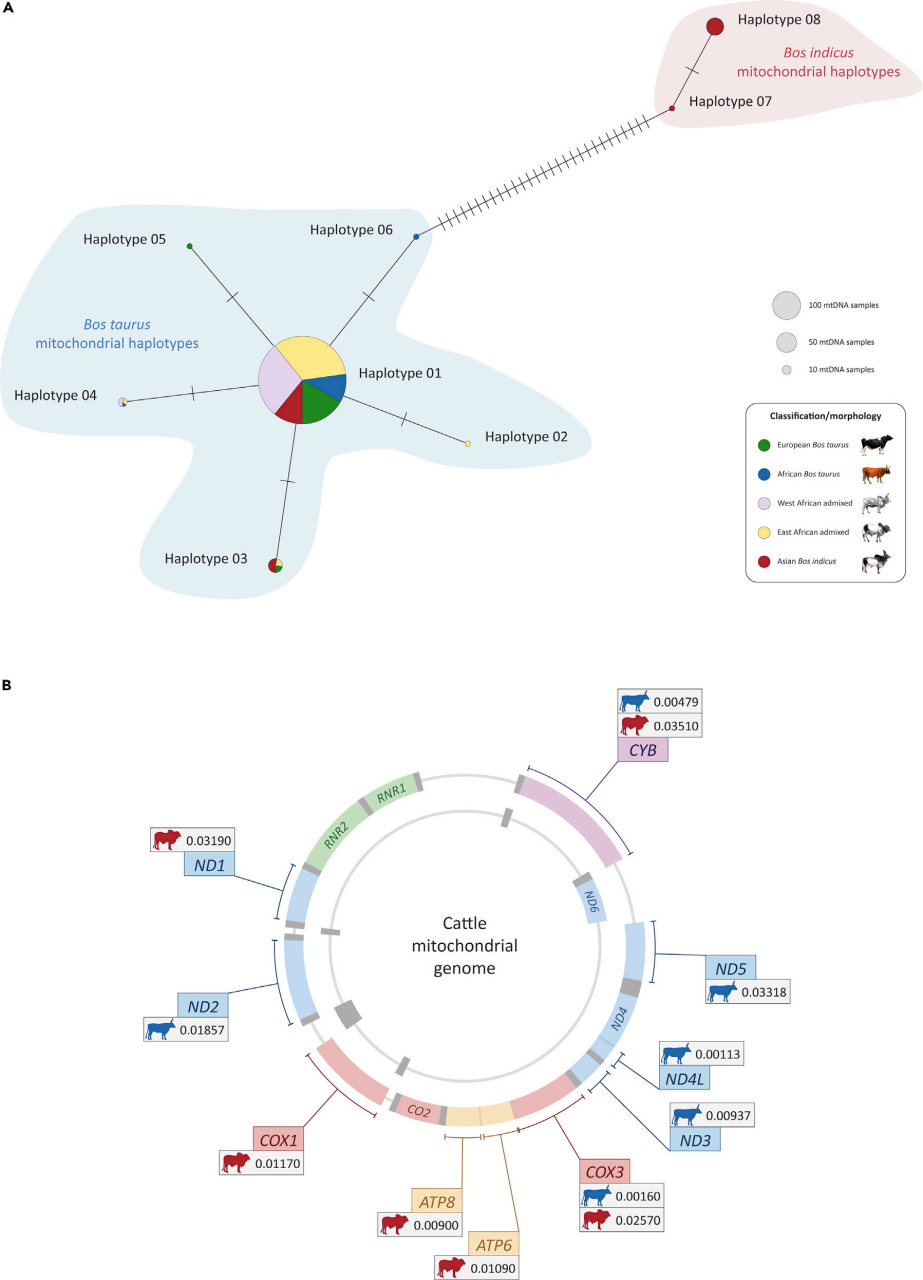
Haplotype diversity and molecular evolution of the cattle mitochondrial genome (A) Network of 491 cattle mtDNA haplotypes generated using 39 ancestry-informative mtDNA SNPs. This mtDNA haplotype network demonstrates that all surveyed African cattle (47 taurine, 156 East African admixed, and 136 West African admixed) possess *Bos taurus* mitochondrial genomes. (B) Evidence for positive selection of protein-coding genes in the cattle mitochondrial genome. The significant p values (<0.05) shown in the gene callouts were obtained using the branch-site test of positive selection at the OXPHOS protein genes. *B. taurus* and *Bos indicus* values are shown with blue and red cattle icons, respectively. The mitochondrially encoded 12 and 16S RNA genes (*RNR1* and *RNR2*) are also shown in green (some figure components created with a BioRender.com license).

After filtering of the 346 mtDNA SNPs on the BovineHD 777K BeadChip and identification of ancestry-informative SNPs that distinguish the taurine and zebu mtDNA genomes, a network of eight haplotypes was generated using 39 mtDNA SNPs and a total of 491 cattle (47 African taurine, 82 European taurine, 156 East African admixed, 136 West African admixed, and 70 Asian zebu). Figure 3A shows this haplotype network and demonstrates that all 339 African taurine and admixed cattle surveyed here possess the taurine mitochondrial genome. In this respect, animals with predominantly zebu ancestry and morphology in Africa represent an example of “massively discordant mitochondrial introgression” (Bonnet et al., 2017), most likely as a result of male-mediated gene flow and genetic drift through preferential dissemination of *B. indicus* genetic material by a relatively small number of Asian zebu cattle, most of which were bulls (Bradley et al., 1994; Loftus et al., 1994a). This scenario is strongly supported by the widespread dissemination of the *B. indicus* Y chromosome in African admixed and morphologically taurine cattle populations (Hanotte et al., 2000; Perez-Pardal et al., 2018).

### Evidence for positive selection at taurine and zebu mitochondrial OXPHOS protein genes

To assess whether the fixation of taurine mitochondrial ancestry in African cattle could be influenced by mitonuclear incompatibilities, we tested whether bovid mitochondrial sequences possess signals of species-specific adaptation. To do this, we obtained high-quality full mtDNA sequences from public DNA sequence databases for 126 African taurine and 21 Asian zebu mitochondrial genomes and 25 mitochondrial genomes for animals from six additional *Bos* species (*B. gaurus* – gaur; *B. frontalis* – mithun; *B. grunniens* – domestic yak; *B. mutus* – wild yak; *B. javanicus* – banteng; and *B. primigenius* – aurochs) (Table S5). Fixed nucleotide substitutions were identified and cataloged from alignments of the 13 mitochondrial OXPHOS protein gene sequences for African taurine vs. Asian zebu, African taurine vs. a range of *Bos* species, and Asian zebu vs. a range of *Bos* species (Table S2).

We further tested for positive selection at the 13 OXPHOS protein genes using the branch-site test of positive selection (Yang and Nielsen, 2002; Zhang et al., 2005) based on the nonsynonymous/synonymous rate ratio (*ω* = *dN*/*dS*) with positive selection indicated by *ω* > 1 (Table S3). Individual genes showing statistically significant evidence for positive selection are indicated in Figure 3B, which shows that eight of the 13 OXPHOS protein genes have been subjected to positive selection in either the taurine (*CYB*, *ND1*, *ND2*, *ND3*, *ND4L*, and *ND5*) or the zebu (*ATP6*, *ATP8*, and *COX1*) mitochondrial genomes and that two (*COX3* and *CYB*) have undergone positive selection in both mtDNA lineages. These results provide evidence for positive selection that could lead to functional differences between zebu and taurine mitochondrial DNA sequences. However, to conclusively determine if these functional differences exist, biochemical and structural analyses of taurine and zebu mitochondrial proteins and their cognate nuclear partners will be required (Du et al., 2017; Sharbrough et al., 2017; Wang et al., 2017).

### Nuclear-encoded mitochondrially targeted genes exhibit signatures of coadaptation across admixed African cattle populations

We then assessed whether admixed African cattle populations also preferentially retain taurine ancestry at nuclear genes encoding products targeted to the mitochondrion and those that directly interact with biomolecules produced from the mitochondrial genome. To do this, we reconstructed the local genomic ancestry of East and West African admixed populations, Asian zebu, and African taurine using MOSAIC (Salter-Townshend and Myers, 2019). Three functional subsets of genes were used in this analysis (Table S6): 1) high-confidence “high-mito” genes (HMG) encoding proteins that directly interact with mtDNA-encoded protein subunits in OXPHOS and ribosomal complexes or that have functions in mtDNA replication (136 genes); 2) lower confidence “low-mito” genes (LMG), which encode proteins that localize to the mitochondrion (661 genes) but are not classified as part of the high-mito subset; and 3) “non-mito” genes (NMG) representing the bulk of the mammalian proteome that does not localize to the mitochondrion (16,383 genes). For each admixed population, the taurine and zebu local ancestry estimates were averaged across mitochondrion-targeted genes (the HMG and LMG subsets) and compared to local ancestry estimates from the genomic background (NMG); this produced deviations in taurine local ancestry for each of the three functional gene subsets. We also generated coancestry curve plots using MOSAIC to determine the estimated number of generations since the start of admixture (Figure S2).

**Figure 4:**
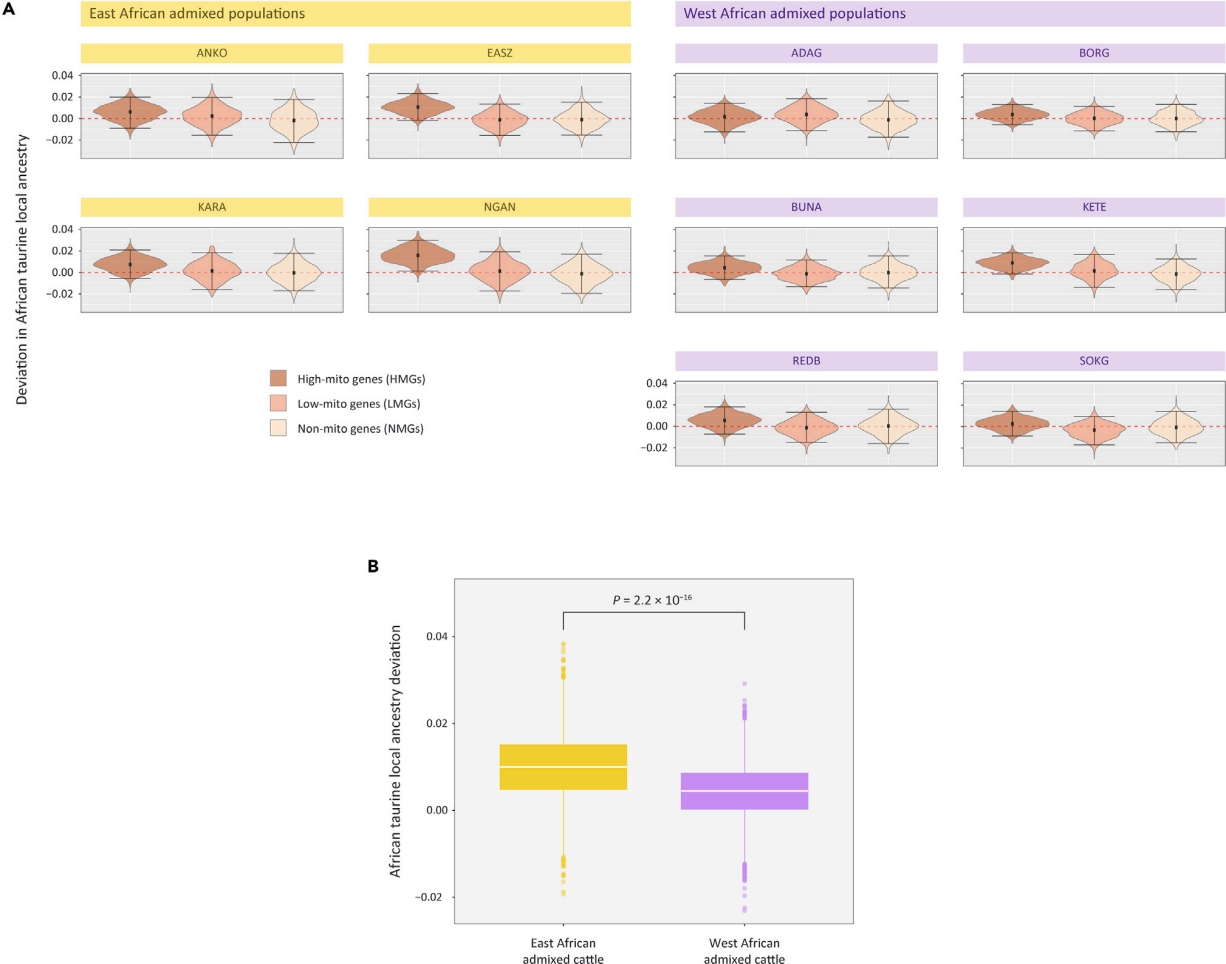
African taurine local ancestry deviations for three different functional gene sets (A) Violin plots of African taurine local ancestry deviations for the HMG, LMG, and NMG subsets with positive deviations indicating retention of African taurine gene haplotypes. Black data points indicate the median values and horizontal lines represent the 95% confidence interval. (B) Boxplot of African taurine local ancestry deviations for the HMG subset in the East African and the West African admixed groups. White lines indicate the median values and yellow and purple boxes indicate the interquartile ranges.

From the bootstrap analysis (Figure 4A), we found that three of the ten African admixed breeds individually exhibit significantly more taurine ancestry for the HMG subset: NGAN (p = 0.0160), KETE (p = 0.0410), and EASZ (p = 0.0430). Using the nonparametric Wilcoxon signed-rank test across the ten admixed African populations, we also demonstrated that the HMG subset exhibited significant differences in mean taurine ancestries compared to the LMG subset (p = 0.0039) and the NMG subset (p = 0.0020). We also compared mean taurine ancestries for the LMG versus the NMG subsets; however, this did not produce a significant statistical test result (p = 0.2754).

### Functional consequences of mitonuclear incompatibilities in admixed African cattle breeds

Previous studies have examined subchromosomal admixture and local ancestry in hybrid taurine/zebu animals (Barbato et al., 2020; Chen et al., 2018; Koufariotis et al., 2018; Mbole-Kariuki et al., 2014; McTavish and Hillis, 2014), and we extend this work to mitonuclear incompatibilities and coadaptation in admixed cattle populations. Using a high-density SNP genotyping array, ten different breeds were examined with genome-wide zebu ancestries ranging between 37% (Borgou) and 74% (Karamojong) and estimated dates for the start of admixture in each population extending from the 14^th^ to the 20^th^ century (Figure S1 and Table S1). A consistent pattern of mitonuclear disequilibria was observed for the functional HMG subset within three breeds of admixed African cattle (EASZ, KETE, and NGAN) (Figure 4A): African taurine local ancestry was uniformly higher for nuclear genes encoding proteins that directly engage with mitochondrial-encoded gene products to form multi-subunit complexes or that directly interact with mitochondrial DNA or RNAs; this subset encompasses genes that encode OXPHOS subunits, ribosomal proteins, tRNA synthetases, and DNA and RNA polymerases. In support of the hypothesis that functional incompatibilities exist between the taurine and zebu mitochondrial genomes, we also find compelling evidence that the two mtDNA lineages have been subjected to positive selection at ten of the 13 OXPHOS protein genes (Figure 3B and Table S2).

These results add support to the findings of Kwon et al. (2022), where they propose that the genomic composition of African admixed cattle has been influenced by selection pressure against the *Bos* indicus mitochondrion. Similarly, although the source population divergence is substantially less in admixed humans, these results are comparable to those obtained by Zaidi and Makova (2019), which support the hypothesis that mitonuclear incompatibilities can act as a driver of selection in admixed human populations. They observed significant enrichment of sub-Saharan African ancestry for HMG subset genes in an African American population with sub-Saharan African and European nuclear ancestry and predominantly sub-Saharan African mtDNA haplotypes. They also observed significant enrichment of Native American ancestry at HMG subset genes in a Puerto Rican population with Native American and European nuclear ancestry and predominantly Native American mtDNA haplotypes.

The functional HMG and LMG subsets containing 136 and 661 genes, respectively (Table S6), were used in the present study for the purpose of evaluating mitonuclear incompatibilities in admixed African cattle populations. However, it is also instructive to examine these genes in the context of recently published high-resolution surveys of African cattle genomic diversity and signatures of selection (Table S4). Some of the genes detected using selection scans and analysis of population differentiated copy number variation in admixed African cattle have biochemical functions and physiological outcomes that may be impacted by mitonuclear incompatibilities (Jang et al., 2021). Proteins encoded by zebu alleles at these nuclear loci and the proteins encoded by the taurine mitochondrial genome may interact suboptimally. For example, the aspartyl-tRNA synthetase 2, mitochondrial gene (*DARS2*), and an HMG subset gene on BTA16, are in the region encompassed by selective sweeps detected separately in the EASZ breed and a composite sample of East African zebu cattle (Bahbahani et al., 2017; Taye et al., 2018). Inspection of the Cattle Gene Atlas (Fang et al., 2020) demonstrates that *DARS2* is most highly expressed in spermatozoa and therefore functionally linked to sperm motility, which may provide an explanation for mitonuclear coevolution in admixed cattle at this locus. In other words, mismatch between proteins encoded by zebu alleles of the *DARS2* gene and the taurine mitochondrial OXPHOS complex proteins could reduce male fertility and lead to positive selection for taurine *DARS2* alleles in admixed populations. In addition, the mitochondrial ribosomal protein S33 gene (*MRPS33*), another HMG subset gene, was detected within a positively selected region on BTA4 when African cattle were compared to commercial European and Asian breeds (Kim et al., 2017) and in analyses of selective sweeps focused on the evolution of thermotolerance in African cattle populations (Taye et al., 2017). Again, in this case, we can hypothesize that incompatibilities exist between zebu *MRPS33* alleles and taurine mitochondrial genes. This could impact metabolism, homeostasis, and heat tolerance, giving rise to selection pressure acting to increase taurine ancestry at the *MRPS33* locus.

Agriculture in Sub-Saharan Africa relies on a diverse array of indigenous cattle breeds, many of which show marked resilience to harsh environments, climatic extremes, and infectious disease—adaptations that have been shaped by their dual taurine-zebu ancestry. Cattle breeding programs in Africa are currently poised to leverage this composite ancestry through genomic selection as a leapfrog technology to bypass conventional breeding to enhance resilience (e.g., via the superior thermotolerance of zebu cattle), production, health, and welfare traits and ultimately improve the livelihoods of smallholder farmers (Ibeagha-Awemu et al., 2019; Marshall et al., 2019; Mrode et al., 2019). Future genetic improvement programs in African cattle will therefore need to consider mitonuclear incompatibilities that could reduce the fitness of hybrid taurine/zebu breeds. Understanding these incompatibilities in hybrid cattle may also provide useful information for targeted editing of both the bovine mitochondrial genome and mitochondrially targeted genes in the nuclear genome (Klucnika and Ma, 2020; Tang et al., 2021). Finally, our results demonstrate that admixed African cattle populations can serve as comparative model systems for understanding the phenotypic consequences of mitonuclear interactions and adaptive and maladaptive genomic introgression in other mammals, including humans.

### Limitations of the study

Although we provide support for the hypothesis that mitonuclear coevolution exists between the nuclear and mitochondrial genomes of hybrid African cattle populations, this work is necessarily limited by the number of populations sampled and the density of the SNP data used. In addition, the genome-wide approach we used here is not directly amenable to gene-by-gene analyses, which could use whole-genome sequence datasets from large numbers of hybrid animals to directly identify incompatibilities between individual nuclear- and mitochondrial-encoded proteins.

## STAR★Methods

### Key resources table

**Table:** 

REAGENT or RESOURCE	SOURCE	IDENTIFIER
**Biological samples**		

*Bos taurus x Bos indicus* (Borgou) [Female, Adult]	TCD DNA Repository	GM01
*Bos taurus x Bos indicus* (Borgou) [Male, Adult]	TCD DNA Repository	GM02
*Bos taurus x Bos indicus* (Borgou) [Male, Adult]	TCD DNA Repository	GM03
*Bos taurus x Bos indicus* (Borgou) [Female, Adult]	TCD DNA Repository	GM04
*Bos taurus x Bos indicus* (Borgou) [Male, Adult]	TCD DNA Repository	GM05
*Bos taurus x Bos indicus* (Borgou) [Female, Adult]	TCD DNA Repository	GM06
*Bos taurus x Bos indicus* (Borgou) [Female, Adult]	TCD DNA Repository	GM07
*Bos taurus x Bos indicus* (Borgou) [Female, Adult]	TCD DNA Repository	GM08
*Bos taurus x Bos indicus* (Borgou) [Female, Adult]	TCD DNA Repository	GM09
*Bos taurus x Bos indicus* (Borgou) [Female, Adult]	TCD DNA Repository	GM10
*Bos taurus x Bos indicus* (Borgou) [Female, Adult]	TCD DNA Repository	GM11
*Bos taurus x Bos indicus* (Borgou) [Female, Adult]	TCD DNA Repository	GM12
*Bos taurus x Bos indicus* (Borgou) [Female, Adult]	TCD DNA Repository	GM13
*Bos taurus x Bos indicus* (Borgou) [Female, Adult]	TCD DNA Repository	GM14
*Bos taurus x Bos indicus* (Borgou) [Female, Adult]	TCD DNA Repository	GM15
*Bos taurus x Bos indicus* (Borgou) [Male, Adult]	TCD DNA Repository	GM16
*Bos taurus x Bos indicus* (Borgou) [Male, Adult]	TCD DNA Repository	GM17
*Bos taurus x Bos indicus* (Borgou) [Male, Adult]	TCD DNA Repository	GM18
*Bos taurus x Bos indicus* (Borgou) [Male, Adult]	TCD DNA Repository	GM19
*Bos taurus x Bos indicus* (Borgou) [Male, Adult]	TCD DNA Repository	GM20
*Bos taurus x Bos indicus* (Borgou) [Male, Adult]	TCD DNA Repository	GM21
*Bos taurus x Bos indicus* (Borgou) [Male, Adult]	TCD DNA Repository	GM22
*Bos taurus x Bos indicus* (Borgou) [Male, Adult]	TCD DNA Repository	GM23
*Bos taurus x Bos indicus* (Borgou) [Male, Adult]	TCD DNA Repository	GM24
*Bos taurus x Bos indicus* (Borgou) [Male, Adult]	TCD DNA Repository	GM25
*Bos taurus x Bos indicus* (Borgou) [Female, Adult]	TCD DNA Repository	GM26
*Bos taurus x Bos indicus* (Borgou) [Female, Adult]	TCD DNA Repository	GM27
*Bos taurus x Bos indicus* (Borgou) [Female, Adult]	TCD DNA Repository	GM28
*Bos taurus x Bos indicus* (Borgou) [Female, Adult]	TCD DNA Repository	GM29
*Bos taurus x Bos indicus* (Borgou) [Female, Adult]	TCD DNA Repository	GM30
*Bos taurus x Bos indicus* (Borgou) [Female, Adult]	TCD DNA Repository	GM31
*Bos taurus x Bos indicus* (Borgou) [Female, Adult]	TCD DNA Repository	GM32
*Bos taurus x Bos indicus* (Borgou) [Female, Adult]	TCD DNA Repository	GM33
*Bos taurus x Bos indicus* (Borgou) [Female, Adult]	TCD DNA Repository	GM34
*Bos taurus x Bos indicus* (Borgou) [Female, Adult]	TCD DNA Repository	GM35
*Bos taurus x Bos indicus* (Borgou) [Female, Adult]	TCD DNA Repository	GM36
*Bos taurus x Bos indicus* (Borgou) [Female, Adult]	TCD DNA Repository	GM37
*Bos taurus x Bos indicus* (Borgou) [Female, Adult]	TCD DNA Repository	GM38
*Bos taurus x Bos indicus* (Borgou) [Male, Adult]	TCD DNA Repository	GM39
*Bos taurus x Bos indicus* (Borgou) [Male, Adult]	TCD DNA Repository	GM40
*Bos taurus x Bos indicus* (Borgou) [Male, Adult]	TCD DNA Repository	GM41
*Bos taurus x Bos indicus* (Borgou) [Male, Adult]	TCD DNA Repository	GM42
*Bos taurus x Bos indicus* (Borgou) [Male, Adult]	TCD DNA Repository	GM43
*Bos taurus x Bos indicus* (Borgou) [Male, Adult]	TCD DNA Repository	GM44
*Bos taurus x Bos indicus* (Borgou) [Male, Adult]	TCD DNA Repository	GM45
*Bos taurus x Bos indicus* (Borgou) [Male, Adult]	TCD DNA Repository	GM46
*Bos taurus x Bos indicus* (Borgou) [Male, Adult]	TCD DNA Repository	GM47
*Bos taurus x Bos indicus* (Borgou) [Male, Adult]	TCD DNA Repository	GM48
*Bos taurus x Bos indicus* (Borgou) [Male, Adult]	TCD DNA Repository	GM49
*Bos taurus x Bos indicus* (Borgou) [Male, Adult]	TCD DNA Repository	GM50
*Bos taurus* (N’Dama) [Female, Adult]	TCD DNA Repository	GM51
*Bos taurus* (N’Dama) [Male, Adult]	TCD DNA Repository	GM52
*Bos taurus* (N’Dama) [Male, Adult]	TCD DNA Repository	GM53
*Bos taurus* (N’Dama) [Male, Adult]	TCD DNA Repository	GM54
*Bos taurus* (N’Dama) [Male, Adult]	TCD DNA Repository	GM55
*Bos taurus* (N’Dama) [Male, Adult]	TCD DNA Repository	GM56
*Bos taurus* (N’Dama) [Male, Adult]	TCD DNA Repository	GM57
*Bos taurus* (N’Dama) [Female, Adult]	TCD DNA Repository	GM58
*Bos taurus* (N’Dama) [Female, Adult]	TCD DNA Repository	GM59
*Bos taurus* (N’Dama) [Male, Adult]	TCD DNA Repository	GM60
*Bos taurus* (N’Dama) [Male, Adult]	TCD DNA Repository	GM61
*Bos taurus* (N’Dama) [Female, Adult]	TCD DNA Repository	GM62
*Bos taurus* (N’Dama) [Female, Adult]	TCD DNA Repository	GM63
*Bos taurus* (N’Dama) [Female, Adult]	TCD DNA Repository	GM64
*Bos taurus* (N’Dama) [Female, Adult]	TCD DNA Repository	GM65
*Bos taurus* (N’Dama) [Male, Adult]	TCD DNA Repository	GM66
*Bos taurus* (N’Dama) [Male, Adult]	TCD DNA Repository	GM67
*Bos taurus* (N’Dama) [Male, Adult]	TCD DNA Repository	GM68
*Bos taurus* (N’Dama) [Male, Adult]	TCD DNA Repository	GM69
*Bos taurus* (N’Dama) [Male, Adult]	TCD DNA Repository	GM70
*Bos taurus* (N’Dama) [Male, Adult]	TCD DNA Repository	GM71
*Bos taurus* (N’Dama) [Male, Adult]	TCD DNA Repository	GM72
*Bos taurus* (N’Dama) [Male, Adult]	TCD DNA Repository	GM73

**Deposited data**		

Raw Genotype Data (BovineHD 777K Array)	This study	Mendeley Data: https://doi.org/10.17632/yt3tgpt48d.1
Raw Genotype Data (BovineHD 777K Array)	Bahbahani et al. (2017)	https://doi.org/10.3389/fgene.2017.00068
Raw Genotype Data (BovineHD 777K Array)	Verdugo et al. (2019)	http://doi.org/10.1126/science.aav1002
Raw mtDNA sequences	See Table S5	See Table S5

**Software and algorithms**		

PLINK v1.9	Chang et al. (2015)	https://www.cog-genomics.org/plink
fastSTRUCTURE v1.0	Raj et al. (2014)	https://rajanil.github.io/fastStructure
Infocalc v1.1	Rosenberg (2005)	https://rosenberglab.stanford.edu/infocalc.html
fastPHASE v1.4	Scheet and Stephens (2006)	https://stephenslab.uchicago.edu/software.html
PAML v4.9	Yang (2007)	https://bioweb.pasteur.fr/packages/pack@paml@4.9e
MOSAIC v1.3.7	Salter-Townshend and Myers. (2019)	https://maths.ucd.ie/∼mst/MOSAIC
SHAPEIT v2	Delaneau et al. (2012)	https://mathgen.stats.ox.ac.uk/genetics_software/shapeit/shapeit.html
BEDTools v2.1.8	Quinlan and Hall (2010)	https://bedtools.readthedocs.io/en/latest
R v3.6.2	R Core Team (2019)	https://www.r-project.org
ggplot2 v.3.3.3	Wickham (2016)	https://ggplot2.tidyverse.org
POPART v1.7	Leigh and Bryant (2015)	http://popart.otago.ac.nz/index.shtml

### Resource availability

#### Lead contact

Further information and inquiries about code, reagents and/or data details may be directed to the lead contact, David E. MacHugh (david.machugh@ucd.ie).

#### Materials availability

This study did not generate new unique reagents.

### Experimental model and subject details

#### Animal sampling

For the present study, new BovineHD 777K BeadChip SNP datasets were generated for 73 adult animals (50 Borgou and 23 N’Dama), of which 41 were male and 32 were female, from DNA samples that were previously published by our group as part of microsatellite-based surveys of cattle genetic diversity in the early 1990s (Freeman et al., 2004; MacHugh et al., 1997). This animal sampling work was completed prior to the requirement for formal Institutional Permission in Ireland, which is based on European Union Directive 2010/63/EU; however, all efforts were made to ensure ethical handling of the animal subjects.

### Method details

#### Animal genotyping

High-density genome-wide SNP array data sets (Illumina® BovineHD 777K BeadChip) corresponding to a total of 456 animals were obtained from published studies (Bahbahani et al., 2017; Verdugo et al., 2019) and for 76 animals previously genotyped by Acceligen. New SNP genotype datasets (50 Borgou and 23 N’Dama) were generated by Weatherbys Scientific (Co. Kildare, Ireland) using standard procedures for Illumina SNP array genotyping. In total, 18 different breeds/populations were represented (Table 1), including two West African taurine breeds (Muturu and N’Dama); two European taurine breeds (Holstein-Friesian and Jersey); ten West and East African admixed zebu-taurine (Adamawa Gudali, Ankole, Borgou, Bunaji, East African Shorthorn Zebu, Karamojong, Keteku, Nganda, Red Bororo, and Sokoto Gudali); and four zebu breeds of South Asian origin (Gir, Hariana, Nelore, and Sahiwal). Table 1 also shows the three- or four-letter codes used to designate each breed.

#### SNP data formatting and quality control

BovineHD 777K SNP locations were remapped to the current bovine genome assembly ARS-UCD1.2 (Rosen et al., 2020) using coordinates from the NAGRP Data Repository as described by Schnabel (2018) and SNP genotype data were merged using PLINK v1.9 (Chang et al., 2015). Quality control (QC) of the combined SNP genotype dataset was also performed using PLINK v1.9 and autosomal SNPs with a call rate <95% and a minor allele frequency (MAF) of <0.05 were filtered from the data.

#### Principal component and structure analyses

Principal component analysis (PCA) of individual animal SNP genotype data for the African taurine (MUTU and NDAG), the East and West African admixed (ADAG, ANKO, BORG, BUNA, EASZ, KARA, KETE, NGAN, REDB, and SOKG) and four Asian indicine (GIR, NELO, SAHI, and HARI) populations was performed using PLINK v1.9 and the results were plotted using ggplot2 v3.3.3 (Wickham, 2016) in the R v3.6.2 environment for statistical computation and graphics (R Core Team, 2019). The genetic structure of each population was also estimated using fastSTRUCTURE v1.0 with *K* = 3 unsupervised modeled ancestries to determine mean African taurine, European taurine, and Asian zebu contributions (Raj et al., 2014).

#### Mitochondrial DNA haplogroup determination

The BovineHD 777K BeadChip includes 346 SNPs located in the mitochondrial genome, which can be used to construct haplotypes and catalog and distinguish the mitochondrial haplogroups characteristic of *B. taurus* and *B. indicus* cattle lineages. For this analysis, the European JRSY and HOLS taurine breeds and the Indo-Pakistan HARI and SAHI Asian indicine breeds were also included to ensure good representation of the *B. taurus* and *B. indicus* mtDNA haplogroups—the ‘T’ and ‘I’ groups, respectively (Chen et al., 2010; Troy et al., 2001). The mtDNA SNPs were filtered using PLINK v1.9 (Chang et al., 2015) such that SNPs with a MAF of <0.10, and a call rate of <95% were removed. Individual animals with a genotype missingness of >95% were also removed. Following this, the most ancestry informative mtDNA SNPs were identified using infocalc v1.1 (Rosenberg, 2005; Rosenberg et al., 2003), which provides *I*_n_, a general measure of the informativeness of an SNP for ancestry assignment. The 50 top ranked SNPs, based on *I*_n_, were then used to generate mtDNA haplotypes with the fastPHASE v1.4 program (Scheet and Stephens, 2006). Haplotype networks were constructed using the POPART v1.7 package (Leigh and Bryant, 2015).

### Quantification and statistical analyses

#### Molecular evolution of mtDNA OXPHOS genes

Complete mitochondrial genome sequences for three groups of cattle and related species were obtained from publicly available DNA sequence databases (Table S5). The mitochondrial genome sequences used represented the African *B. taurus* (126 animals), and Asian *B. indicus* (21 animals) mtDNA lineages, and the following additional *Bos* species: *B. gaurus* – gaur (6 animals); *B. frontalis* – mithun (4 animals); *B. grunniens* – domestic yak (5 animals); *B. mutus* – wild yak (4 animals); *B. javanicus* – banteng (4 animals); *and B. primigenius* – aurochs (2 animals). The protein-coding sequence for 13 essential OXPHOS genes were aligned using the MAFFT v.7.49 software package (Katoh et al., 2019). Evidence for positive selection at the 13 OXPHO protein genes (*ATP6*, *ATP8*, *CYB*, *COX1*, *COX2*, *COX3*, *ND1*, *ND2*, *ND3*, *ND4*, *ND4L*, *ND5*, and *ND6*) was evaluated using the *d*_N_/*d*_S_ ratio (*ω*) branch site test for positive selection (Yang and Nielsen, 2002; Zhang et al., 2005) with the CODEML branch-site models MA(ω > 1) vs. MA(ω = 1) implemented in the PAML v4.9 software package (Yang, 2007).

#### Local ancestry analysis of admixed populations

Local ancestry across the bovine genome for each African admixed breed (ADAG, ANKO, BORG, BUNA, EASZ, KARA, KETE, NGAN, REDB, and SOKG) was inferred using MOSAIC v1.3.7 (Salter-Townshend and Myers, 2019). The MOSAIC algorithm, unlike other methods, does not require defined surrogate donor reference populations for the mixing ancestral populations; it fits a two-layer Hidden Markov Model (HMM) that determines how closely related each segment of chromosome in each admixed individual genome is to the segments of chromosomes in individual genomes from potential donor populations. While determining local ancestry along each chromosome, MOSAIC also infers the number of generations since the admixture process started for a particular population. The potential donor populations used for the MOSAIC local ancestry analysis were the two West African *B. taurus* breeds (MUTU and NDAG) and two of the Asian *B. indicus* breeds (GIR and NELO). The MOSAIC algorithm requires phased haplotypes and a recombination rate map; therefore, SHAPEIT v2 (r900) (Delaneau et al., 2012) was used to generate phased haplotypes and a published cattle recombination map was employed (Ma et al., 2015).

#### Detection of taurine local ancestry deviation

To determine if there was significant retention of *B*. *taurus* nuclear genes that encode mitochondrially targeted proteins (*Nu-mito* genes) in African admixed cattle, we used an approach modified from surveys of mitonuclear incompatibilities in modern admixed human populations (Sloan et al., 2015; Zaidi and Makova, 2019) and a study of ancient gene flow between archaic hominins (*H. neanderthalensis* and *H. denisova*) and modern humans (Sharbrough et al., 2017). Firstly, the MitoCarta 2.0 database resource (Calvo et al., 2016) was used to obtain an inventory of genes that produce the nuclear-encoded component of the mammalian mitochondrial proteome, i.e., proteins with experimental evidence for localization in the mitochondrion. Following this, the Ensembl BioMart tool (Yates et al., 2020) was used to generate a list of 1158 bovine Nu-mito genes, which was classified into two functional subsets as defined by Sloan et al. (2015) and also used by Sharbrough et al. (2017) and Zaidi and Makova (2019). These subsets were denoted as 1) high-confidence “*high-mito*” genes (HMG) encoding proteins that directly interact with mtDNA-encoded protein subunits in OXPHOS and ribosomal complexes, or that have functions in mtDNA replication (136 genes); and 2) lower confidence “*low-mito*” genes (LMG), which encode proteins that localize to the mitochondrion (661 genes) but are not classified as part of the high-mito subset. Finally, a third group of “*non-mito*” genes (NMG) was generated, which includes the bulk of the mammalian proteome that does not localize to the mitochondrion (16,383 genes). Table S6 provides further detail for the functional gene subsets used to detect evidence for mitonuclear incompatibilities in African admixed cattle populations.

The local ancestry estimates generated using MOSAIC for each SNP across the genome were cataloged and the BEDTools v2.18 software suite (Quinlan and Hall, 2010) was then used to intersect these SNPs with windows spanning 2.5 Mb upstream and downstream of genes within each of the three functional gene subsets. Following this, and as described by Zaidi and Makova (2019), for each of the three subsets an unweighted block bootstrap approach was used to generate distributions of local ancestry deviation toward more or less *B. taurus* ancestry. The first step in this methodology is subtraction of the mean ancestry fraction across the local ancestry estimate for each SNP (the *expectation*), which produces the deviation in local ancestry at each SNP locus. For each functional gene subset, the number of windows sampled with replacement was the same as the number of HMG subset genes (n = 136). In each case, the mean ancestry deviation was estimated and then averaged across all windows. Bootstrap resampling (1000 replicates) was used to generate a distribution of mean deviations in local ancestry for each of the three functional gene subsets. Overall significance of the distributions was assessed by the proportion of the distribution that overlapped zero. Mean taurine ancestry was determined for each of the gene subsets across all ten populations. Differences in the population means of these were assessed using the non-parametric Wilcoxon signed-rank test in R v3.6.2 (R Core Team, 2019).

## Data Availability

• New and previously unpublished Illumina BovineHD 777K BeadChip SNP data used for this study have been deposited in the Mendeley Data repository; the DOI is listed in the Key resources table. • This paper does not report original code. • Any additional information required to reanalyse the data reported in this paper is available from the Lead contact.

## Supplemental information

Document S1. Figures S1 and S2 and Tables S1–S4

Table S5: Bos species mitochondrial genome sequences used for molecular evolution analysis, related to STAR Methods

Table S6: Functional gene subsets used to detect evidence for mitonuclear disequilibria in African admixed cattle populations, related to STAR Methods
