# Supplementary material for: Genome-wide local ancestry and evidence for mitonuclear coadaptation in African hybrid cattle populations
Source: iScience. 2022 Jun 26;25(7):104672. doi: 10.1016/j.isci.2022.104672 (PMC9272374; doi:10.1016/j.isci.2022.104672)
Supplement: Document S1. Figures S1 and S2 and Tables S1–S4 [file mmc1.pdf]

iScience, Volume *25*

Supplemental information

Genome-wide local ancestry and evidence for mitonuclear coadaptation in African hybrid cattle populations

James A. Ward, Gillian P. McHugo, Michael J. Dover, Thomas J. Hall, Said Ismael Ng'ang'a, Tad S. Sonstegard, Daniel G. Bradley, Laurent A.F. Frantz, Michael Salter-Townshend, and David E. MacHugh

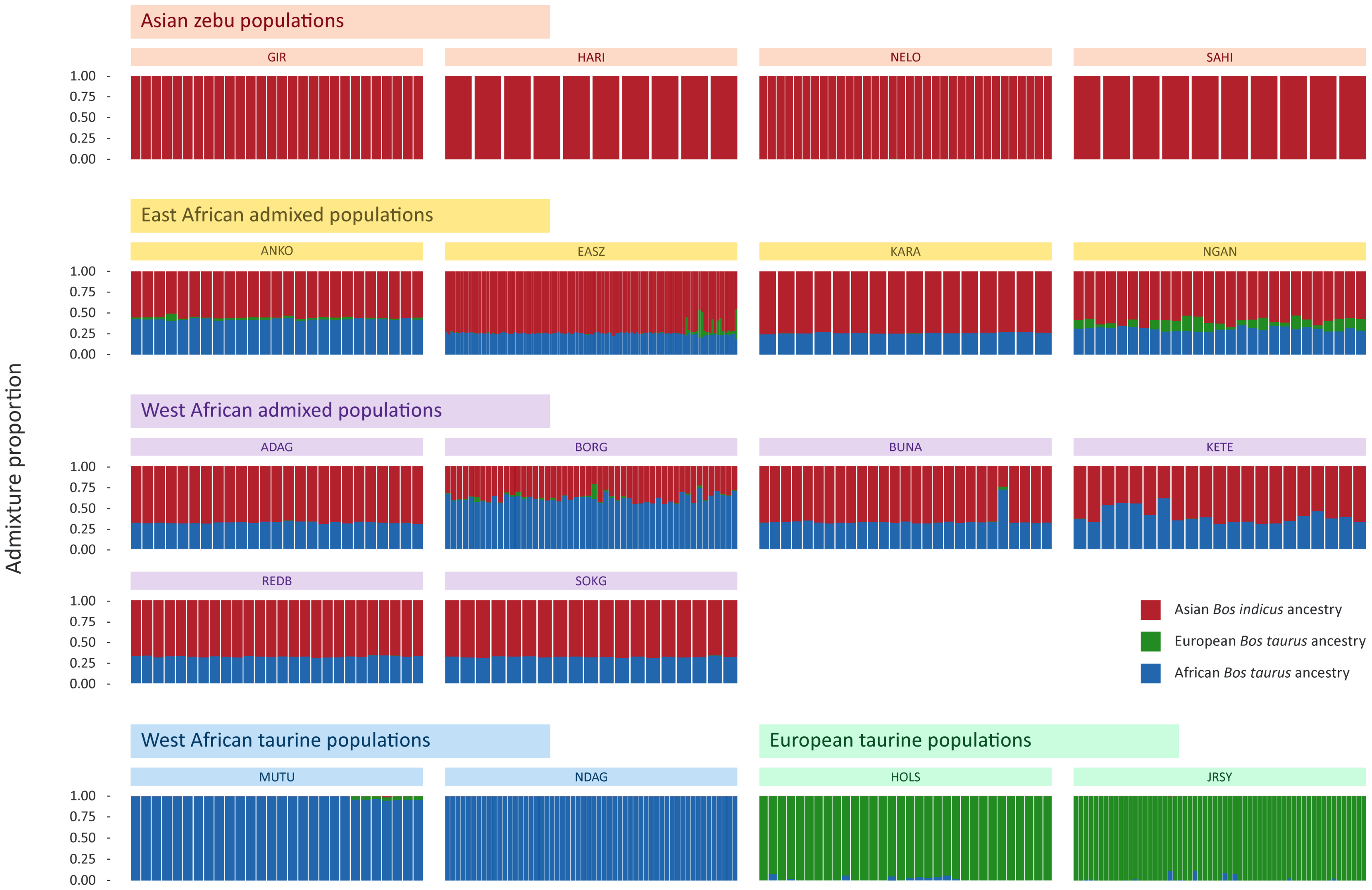

Figure S1. Unsupervised genetic structure plot for Asian zebu, East and West African admixed cattle, and West African and European taurine breeds. Results for an inferred number of ancestry clusters of *K* = 3 is shown, which corresponds to Asian *Bos indicus* (red), European *Bos taurus* (green), and African *B. taurus* (blue) ancestral components, respectively. Related to "Main Figure 2".

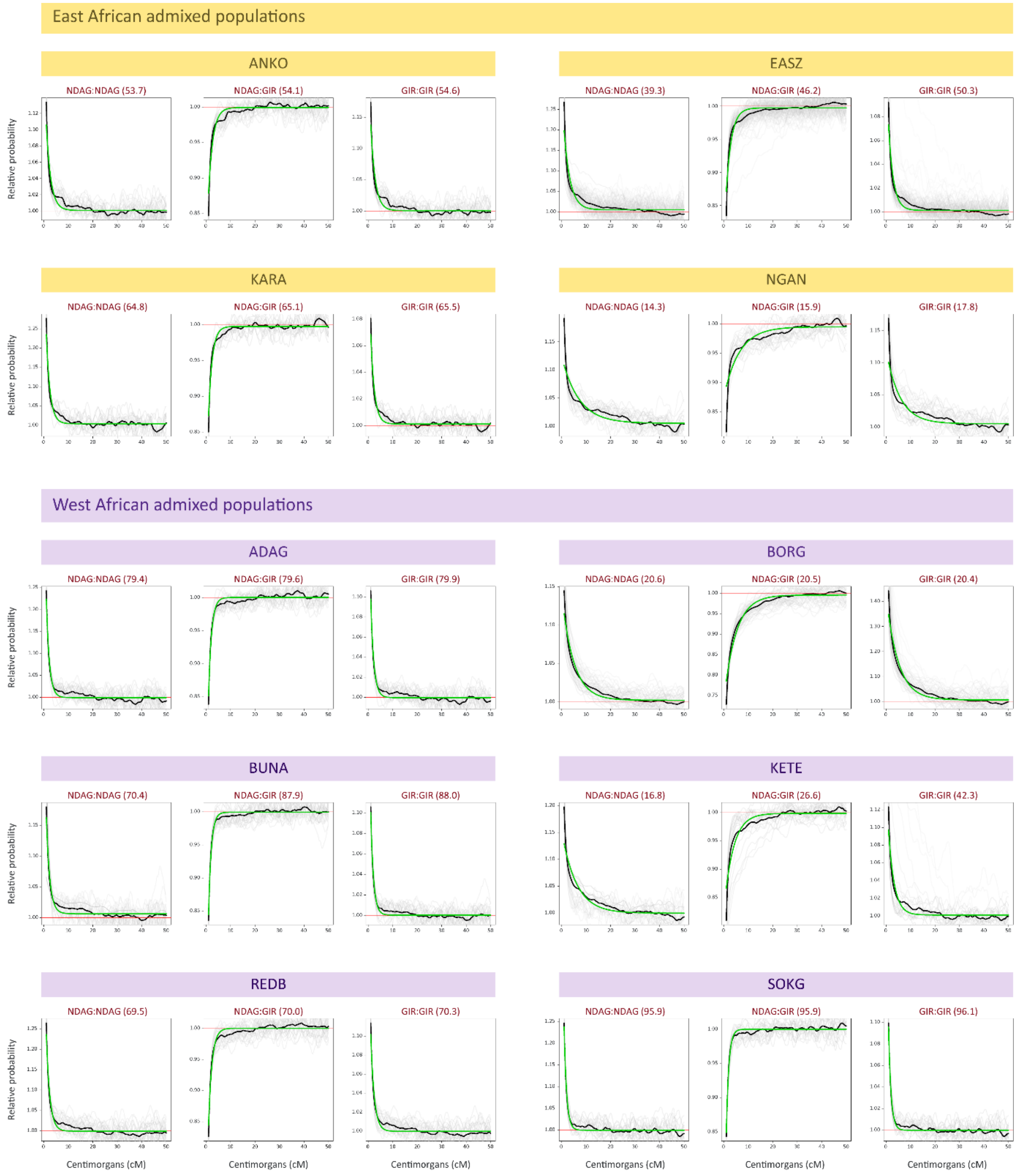

Figure S2. Coancestry curve plots generated using MOSAIC for 10 East and West African admixed cattle populations. These curves show the exponential decay of the ratio of probabilities of pairs of local ancestries (y-axis) as a function of genetic distance (x-axis). The pair of ancestries used for each curve is shown on the top of each plot with the estimated number of generations since the start of admixture in brackets. For each plot, the green line represents the fitted curve, the black line shows the across targets ratio, and the grey lines indicate the per target ratio (further information in Salter-Townshend and Myers, 2019). Related to STAR Methods.

Table S1. Ancestry components estimated using fastSTRUCTURE with estimated times for the start of the admixture process in African admixed cattle populations generated using MOSAIC. Related to STAR Methods.

Code	Type/morphology	Country of origin	African taurine ancestry	European taurine ancestry	Asian zebu ancestry	Generations since start of admixture	Start of admixture
MUTU	West African taurine	Nigeria	0.990 ± 0.019	0.010 ± 0.018	0.000 ± 0.003		----
NDAG	West African taurine	Guinea	1.000 ± 0.000	0.000 ± 0.000	0.000 ± 0.000		----
HOLS	European taurine	Netherlands	0.010 ± 0.025	0.990 ± 0.025	0.000 ± 0.000		----
JRSY	European taurine	United Kingdom	0.010 ± 0.022	0.990 ± 0.022	0.000 ± 0.000		----
ANKO	East African admixed	Uganda	0.420 ± 0.010	0.020 ± 0.016	0.550 ± 0.013	54.1	1641 – 1804 CE
EASZ	East African admixed	Kenya	0.250 ± 0.015	0.020 ± 0.065	0.730 ± 0.055	46.2	1697 – 1835 CE
KARA	East African admixed	Uganda	0.260 ± 0.008	0.000 ± 0.001	0.740 ± 0.008	65.1	1564 – 1760 CE
NGAN	East African admixed	Uganda	0.310 ± 0.024	0.100 ± 0.052	0.590 ± 0.039	15.9	1909 – 1956 CE
ADAG	West African admixed	Nigeria	0.320 ± 0.010	0.000 ± 0.001	0.680 ± 0.010	79.6	1463 – 1702 CE
BORG	West African admixed	Benin	0.610 ± 0.047	0.010 ± 0.027	0.370 ± 0.055	20.5	1877 – 1938 CE

Code	Type/morphology	Country of origin	African taurine ancestry	European taurine ancestry	Asian zebu ancestry	Generations since start of admixture	Start of admixture
BUNA	West African admixed	Nigeria	0.340 ± 0.076	0.000 ± 0.007	0.660 ± 0.083	87.9	1405 – 1668 CE
KETE	West African admixed	Nigeria	0.400 ± 0.093	0.000 ± 0.001	0.600 ± 0.093	26.6	1834 – 1914 CE
REDB	West African admixed	Nigeria	0.320 ± 0.009	0.000 ± 0.001	0.680 ± 0.009	70	1530 – 1740 CE
SOKG	West African admixed	Nigeria	0.320 ± 0.008	0.000 ± 0.000	0.680 ± 0.008	95.9	1349 – 1636 CE
GIR	Asian zebu	India	0.000 ± 0.000	0.000 ± 0.000	1.000 ± 0.000		----
HARI	Asian zebu	India	0.000 ± 0.000	0.000 ± 0.000	1.000 ± 0.000		----
NELO	Asian zebu	Brazil	0.000 ± 0.000	0.000 ± 0.001	1.000 ± 0.001		----
SAHI	Asian zebu	India	0.000 ± 0.000	0.000 ± 0.000	1.000 ± 0.000		----

Note. For each admixed population, the generations since admixture started were obtained from the NDAG:GIR coancestry plots (Fig. S2). A generation interval range of 4–7 years for managed domestic cattle was used. CE = Common Era.

Table S2. Nucleotide substitutions determined from alignments of the protein-coding sequences of 13 mitochondrial OXPHOS protein genes for three groups of Bovinae species/subspecies. The taxa examined included African *Bos taurus*, Asian *Bos indicus* and a range of *Bos* species (*Bos gaurus*, *Bos grunniens*, *Bos javanicus*, *Bos mutus, Bos frontalis, and Bos primigenius*). The "Single" and "Multiple" terms refer to mtDNA sites where a single mutation has occurred (i.e., two variants are present) or where multiple substitutions have produced more than two variants at the same site. Related to STAR Methods.

Gene name	Gene symbol	Length in bp (aligned)	African *B. taurus vs.* Asian *B. indicus*		African *B. taurus vs.* *Bos* species		Asian *B. indicus vs.* *Bos* species	
			Single	Multiple	Single	Multiple	Single	Multiple
ATP synthase 6	*ATP6*	692	8	4	30	8	23	14
ATP synthase 8	*ATP8*	204	5	2	13	4	12	4
Cytochrome b	*CYB*	1158	14	4	45	15	38	19
Cytochrome c oxidase I	*COX1*	1570	3	0	29	0	28	0
Cytochrome c oxidase II	*COX2*	695	2	2	13	2	14	0
Cytochrome c oxidase III	*COX3*	798	4	4	14	12	13	14
NADH dehydrogenase subunit 1	*ND1*	972	8	2	24	6	26	6
NADH dehydrogenase subunit 2	*ND2*	1061	6	2	52	11	51	11
NADH dehydrogenase subunit 3	*ND3*	353	1	2	6	0	4	2
NADH dehydrogenase subunit 4	*ND4*	1400	10	6	52	19	53	22
NADH dehydrogenase subunit 4L	*ND4L*	301	0	2	11	0	10	2
NADH dehydrogenase subunit 5	*ND5*	1891	7	17	114	46	72	56
NADH dehydrogenase subunit 6	*ND6*	536	8	2	22	4	22	3

Table S3. Results for the branch-site test of positive selection (BSPS) for 13 mitochondrial OXPHOS protein gene sequences in three different *Bos* groups. Shown are the number of parameters, degrees of freedom, log-likelihood values, and likelihood ratio test results. Significant *P* values (< 0.05) indicating positive selection for individual genes are shown in bold underline. Related to "Main Figure 3".

Bos taurus mitochondrial genome sequences							
**Gene symbol**	**No. param. H_A**	**No. param. H_0**	**df**	**LnL H_A**	**LnL H_0**	**LRT(2ΔLnL)**	***P*** **(χ^2)**
ATP6	381	380	1	-2101.88	-2101.79	-0.18280	1.00000
ATP8	381	380	1	-527.49	-526.98	-1.01728	1.00000
CYB	381	380	1	-3334.59	-3334.59	0.00004	**<u>0.00479</u>**
COX1	381	380	1	-3955.67	-3955.67	-0.00081	1.00000
COX2	381	380	1	-1696.07	-1696.06	-0.01870	1.00000
COX3	381	380	1	-1774.40	-1774.40	0.00000	**<u>0.00160</u>**
ND1	381	380	1	-2475.83	-2475.83	-0.00070	1.00000
ND2	381	380	1	-3748.60	-3748.60	0.00054	**<u>0.01857</u>**
ND3	381	380	1	-867.15	-867.15	0.00014	**<u>0.00937</u>**
ND4	381	380	1	-3826.87	-3826.87	-0.00067	1.00000
ND4L	381	380	1	-781.98	-781.98	0.00000	**<u>0.00113</u>**
ND5	381	380	1	-5019.63	-5019.63	0.00173	**<u>0.03318</u>**
ND6	381	380	1	-1931.80	-1931.80	-0.00013	1.00000

Bos indicus mitochondrial genome sequences							
**Gene symbol**	**No. param. H_A**	**No. param. H_0**	**df**	**LnL H_A**	**LnL H_0**	**LRT(2ΔLnL)**	**P (χ^2)**
ATP6	381	380	1	-1894.03	-1894.03	0.00019	**0.01090**
ATP8	381	380	1	-526.98	-526.98	0.00013	**0.00900**
CYB	381	380	1	-3334.59	-3334.59	0.00193	**0.03510**
COX1	381	380	1	-3955.67	-3955.67	0.00021	**0.01170**
COX2	381	380	1	-1696.06	-1696.06	-0.00057	1.00000
COX3	381	380	1	-1774.40	-1774.40	0.00104	**0.02570**
ND1	381	380	1	-2475.83	-2475.84	0.00159	**0.03190**
ND2	381	380	1	-3747.84	-3747.84	-0.00085	1.00000
ND3	381	380	1	-866.65	-866.65	-0.00031	1.00000
ND4	381	380	1	-3826.87	-3826.87	-0.00053	1.00000
ND4L	381	380	1	-781.98	-781.98	-0.00041	1.00000
ND5	381	380	1	-5019.63	-5019.63	-0.00374	1.00000
ND6	381	380	1	-1931.80	-1931.80	-0.00007	1.00000

Bos species group mitochondrial genome sequences							
**Gene symbol**	**No. param. H_A**	**No. param. H_0**	**df**	**LnL H_A**	**LnL H_0**	**LRT(2ΔLnL)**	**P (χ^2)**
ATP6	381	380	1	-1894.03	-1894.03	-0.00045	1.00000
ATP8	381	380	1	-527.49	-527.49	-0.00043	1.00000
CYB	381	380	1	-3334.59	-3334.59	0.00042	**0.01639**
COX1	381	380	1	-3955.67	-3955.67	-0.00001	1.00000
COX2	381	380	1	-1696.06	-1696.06	0.00000	1.00000
COX3	381	380	1	-1774.40	-1774.40	-0.00017	1.00000
ND1	381	380	1	-2475.83	-2475.83	0.00027	**0.01311**
ND2	381	380	1	-3748.60	-3748.60	0.00026	**0.01291**
ND3	381	380	1	-867.15	-867.15	-0.00137	1.00000
ND4	381	380	1	-3825.86	-3825.86	-0.00035	1.00000
ND4L	381	380	1	-781.98	-781.98	-0.00001	1.00000
ND5	381	380	1	-5018.53	-5018.53	-0.00080	1.00000
ND6	381	380	1	-1931.80	-1931.80	-0.00076	1.00000

Table S4. HMG and LMG functional subset genes detected in multiple studies (≥ 2) of genomic selective sweeps in African cattle populations. Related to STAR Methods.

Gene symbol	Gene name	Functional subset	Chromosomal location	Citations
ACO2	Aconitase 2 gene	LMG	BTA5	(Bahbahani et al., 2018a; Taye et al., 2018)
BCL2L13	BCL2 like 13 gene	LMG	BTA5	(Taye *et al.*, 2018; Taye et al., 2017)
CA5A	Carbonic anhydrase 5A gene	LMG	BTA18	(Kim et al., 2017; Taye *et al.*, 2018; Taye *et al.*, 2017)
CMC1	C-X9-C motif containing 1 gene	LMG	BTA22	(Bahbahani *et al.*, 2018a; Kim *et al.*, 2017)
CYP24A1	Cytochrome P450, family 24, subfamily A, polypeptide 1 gene	LMG	BTA13	(Bahbahani et al., 2017; Tijjani et al., 2019)
DARS2	Aspartyl-tRNA synthetase 2, mitochondrial gene	HMG	BTA16	(Bahbahani *et al.*, 2017; Taye *et al.*, 2018)
DNAJC11	DnaJ heat shock protein family (Hsp40) member C11 gene	LMG	BTA16	(Bahbahani *et al.*, 2017; Taye *et al.*, 2018; Taye *et al.*, 2017)
HDHD3	Haloacid dehalogenase like hydrolase domain containing 3 gene	LMG	BTA8	(Kim *et al.*, 2017; Taye *et al.*, 2017)
HIBADH	3-hydroxyisobutyrate dehydrogenase gene	LMG	BTA4	(Kim *et al.*, 2017; Taye *et al.*, 2018; Taye *et al.*, 2017)
HIGD1A	HIG1 hypoxia inducible domain family member 1A gene	LMG	BTA22	(Bahbahani *et al.*, 2018a; Jang et al., 2021; Tijjani *et al.*, 2019)
MRPS33	Mitochondrial ribosomal protein S33 gene	HMG	BTA4	(Kim *et al.*, 2017; Taye *et al.*, 2017)
MSRB3	Methionine sulfoxide reductase B3 gene	LMG	BTA4	(Bahbahani *et al.*, 2017; Kim *et al.*, 2017; Taye *et al.*, 2018; Taye *et al.*, 2017)
NGRN	Neugrin, neurite outgrowth associated gene	LMG	BTA21	(Taye *et al.*, 2018; Taye *et al.*, 2017)

PDE12	Phosphodiesterase 12 gene	LMG	BTA22	(Bahbahani *et al.*, 2017; Taye *et al.*, 2018)
PUSL1	Pseudouridine synthase like 1 gene	LMG	BTA16	(Taye *et al.*, 2018; Taye *et al.*, 2017)
SARDH	Sarcosine dehydrogenase gene	LMG	BTA11	(Bahbahani *et al.*, 2018a; Tijjani *et al.*, 2019)
SLC25A11	Solute carrier family 25 member 11 gene	LMG	BTA19	(Bahbahani *et al.*, 2017; Kim *et al.*, 2017; Taye *et al.*, 2018)
SLC25A21	Solute carrier family 25 member 21 gene	LMG	BTA21	(Bahbahani et al., 2018b; Tijjani *et al.*, 2019)
SOD1	Superoxide dismutase 1 gene	LMG	BTA1	(Kim *et al.*, 2017; Taye *et al.*, 2018; Taye *et al.*, 2017)
SUCLG2	Succinate-CoA ligase GDP-forming subunit beta gene	LMG	BTA22	(Jang *et al.*, 2021; Kim et al., 2020)
TIMM21	Translocase of inner mitochondrial membrane 21 gene	LMG	BTA24	(Bahbahani *et al.*, 2018a; Tijjani *et al.*, 2019)
TOMM22	Translocase of outer mitochondrial membrane 22 gene	LMG	BTA5	(Kim *et al.*, 2017; Taye *et al.*, 2017)
YME1L1	YME1 like 1 ATPase gene	LMG	BTA13	(Taye *et al.*, 2018; Taye *et al.*, 2017)
